# Exploring an Existing Weight Management App for Use With Adolescents and Young Adults With Spina Bifida: Usability Study

**DOI:** 10.2196/15153

**Published:** 2019-10-10

**Authors:** Colleen Stiles-Shields, Brittney Garcia, Kimberly Villota, Elicia Wartman, Adrien M Winning, Grayson N Holmbeck

**Affiliations:** 1 Population Behavioral Health Department of Psychiatry and Behavioral Sciences Rush University Medical Center Chicago, IL United States; 2 Department of Psychology Loyola University Chicago Chicago, IL United States

**Keywords:** spina bifida occulta, mHealth, mobile apps, usability testing, adolescent, young adult, weight reduction programs, body weight maintenance

## Abstract

**Background:**

Adolescents and young adults with spina bifida (AYA-SBs) have unique user needs, given their variable and complex symptom profile. Owing to multiple barriers to prevention and intervention treatments for secondary conditions (eg, obesity), AYA-SBs may benefit from the use of behavioral intervention technologies (BITs). However, as BITs are often designed and tested with typically developing individuals, it is unclear if existing BITs may be usable for AYA-SBs.

**Objective:**

This study aimed to evaluate the usability of a high-quality, publicly available, weight management–focused mobile BIT (smartphone app) for AYA-SBs.

**Methods:**

Overall, 28 AYA-SBs attending a Young Men’s Christian Association–based summer camp completed 4 structured usability tasks using a weight management app designed for the general public called My Diet Coach (Bending Spoons). Learnability was measured by (1) time to complete task, (2) number of user errors, and (3) correct entry of data when requested by the app. Satisfaction and general usability were measured via self-reported questionnaires and qualitative feedback following interactions with the app.

**Results:**

The majority of the sample were able to complete the tasks, with increased completion rates and improved times on second attempts of the tasks (*P*s<.05). Errors were common, and discrepancies emerged between quantitative and qualitative feedback such that self-reported measures indicated dissatisfaction but qualitative feedback was generally positive. Suggested improvements to the app included (1) tutorials, (2) simplifying the design, (3) more activity options for those who ambulate by wheelchair, and (4) notifications to prompt use.

**Conclusions:**

AYA-SBs were able to learn how to complete specific tasks independently on a weight management app, but design changes consistent with previously proposed user needs were recommended. Rather than designing entirely new BITs, it may be possible to adapt existing technologies to personalize BITs for specific populations such as AYA-SBs.

## Introduction

### Background

Spina bifida (SB) is the most common congenital birth defect affecting the central nervous system and requires the management of both a complex medical treatment regimen and a variety of cognitive and psychosocial comorbidities [1,2]. Complicating the management of this condition, adolescents and young adults (AYAs) with SB (hereafter referred to as AYA-SBs) are disproportionately impacted by obesity [3]. Beyond the typical risk factors associated with obesity as a secondary condition, obesity poses a greater risk for other outcomes, such as muscle loss, pressure sores, depression, complications of surgery, and decreased social and physical activities [3-5]. In addition, people with SB and obesity are doubly at risk for social rejection, given the combination of 2 visible vulnerabilities: physical disability and obese status [6,7]. Therefore, preventing and reducing obesity in individuals with SB is a critical goal.

Multiple emotional (eg, low motivation and depressive symptoms) and practical barriers (eg, ambulation status and transportation) to addressing obesity and healthy lifestyles have been identified for AYA-SBs [3,5,8-10]. Behavioral intervention technologies (BITs), the use of technology to deliver behavioral health interventions [11], have demonstrated promising effects on health outcomes for pediatric samples [12]. AYA-SBs report using a variety of technology and media [13]. Furthermore, a self-management behavioral intervention technology (BIT) for people with disabilities (ie, SB, cerebral palsy, and spinal cord injuries), Interactive Mobile Health and Rehabilitation (iMHere), has demonstrated feasibility and benefits to high users of the system [14]. Technology usage, in combination with the barriers faced to addressing obesity, implicates the use of BITs as a delivery mechanism for obesity and healthy lifestyle monitoring and interventions for AYA-SBs.

Given their unique user needs, calls for personalizing BITs for people with disabilities have been made [15]. For example, AYA-SBs are unique in that they have symptoms that overlap with chronic medical conditions and physical and intellectual disabilities. For this reason, a user needs model for BITs that support self-management in SB has been created [16]. The model includes (1) behavioral skills– and evidence-based change strategies that avoid abstract concepts and help to categorize behavior, (2) elements that are multisensory (eg, text and audio) and use multiple methods (eg, visualizations and passive data collection), (3) being capable of being used across multiple platforms, (4) linear and user-driven workflows, and (5) expert and/or peer support. However, before using this model to create new BITs designed for AYA-SBs, currently available BITs should be evaluated. This is for multiple reasons. First, although the previously mentioned iMHere platform already exists, it is (1) currently only available for Android devices and (2) designed to promote self-management across a variety of issues pertinent to those with disabilities. AYA-SBs without an Android and/or those who might wish to exclusively focus on an issue such as weight management face barriers to utilizing this BIT. Therefore, AYA-SBs who face such barriers are likely to search the publicly available marketplace for another BIT to suit their needs. Second, given the high number of BITs already available for health and body image management, it is possible that elements of existing BITs may be appropriate for AYA-SBs. Therefore, evaluating how currently available BITs function for AYA-SBs is necessary to potentially avoid *reinventing the wheel* in terms of some BIT tools.

To accomplish the task of evaluating how well currently available BITs may serve this population, usability testing was utilized. Usability testing is the systematic observation of planned tasks by potential end users to improve the design of a product or technology [17]. Learnability, or how well a user can complete tasks during the first interaction(s) on an app [18], was the usability attribute of interest. Indeed, if AYA-SBs independently download a publicly available app, their ability to learn to use the app is essential for correct use. On average and based on evaluations of healthy adults, users report spending about 5 min or less learning how to use a new app [19,20], and AYAs have been found to quickly dismiss technological tools that misalign with their expectations [21,22]. Furthermore, AYA-SBs tend to have difficulties with assembled processing (ie, learning to construct and digest information) [23]. This difficulty means that even the most ideal app (eg, grounded within evidence-based behavioral change theories and high usability) might have different learnability for a user with SB. Therefore, in assessing the usability of BITs for AYA-SBs, how quickly and well the users learn to use the BIT is important.

### Purpose

The purpose of this study was to evaluate the usability attribute of learnability for a high-quality [18], publicly available, weight management–focused mobile BIT (smartphone app) for AYA-SBs. We hypothesized that, in evaluating an app rated as having high quality for typically developing populations [18], the majority of users would be able to complete tasks and do so in 5 min or less by their second attempt of a task [19,20]. However, given the variable symptom and functioning profile of AYA-SBs [16], it was anticipated that high variability across the sample would be observed for all evaluated usability metrics.

## Methods

### Participants

Participants were recruited from the Young Men’s Christian Association (YMCA)-sponsored Camp Independence during the 2018 summer sessions. Camp Independence is located in Illinois (USA) and is a sleep-away camp designed for AYA-SBs. Programming includes (1) a 1-week stay with similarly aged campers, (2) typical camp-based activities (eg, swimming) with accommodations for camper needs, and (3) camp-based interventions to promote medical and social independence [24-26]. Participants were eligible for study inclusion if they (1) had SB, (2) were aged between 13 and 30 years, (3) attended Camp Independence during the summer of 2018, (4) had previously used a mobile app independently, and (5) could read and write in English.

### My Diet Coach

The app that was selected for usability testing was the My Diet Coach app. My Diet Coach was selected as it is (1) highly rated for quality using the Mobile App Rating Scale [18,27], (2) publicly available on both iTunes [28] and the Google Play Store [29], and (3) cost free (a version is also available for purchase). The app includes features such as a meal and activity journal, tips, and user-selected challenges.

### Procedure

This study was approved by the Loyola University Chicago institutional review board. Participants aged 18 years and older provided informed consent. Participants younger than 18 years provided informed assent, and their parents provided consent.

#### Usability Testing

To assess the usability attribute of learnability, the participants were asked to complete specific tasks (eg, log a food item) with an app on a mobile device. These tasks were related to food intake and activity monitoring, which were selected because (1) they are common tasks for weight loss and management and (2) they could be evaluated relatively quickly, so as to not keep participants from their camp activities. Learnability was measured by (1) time to complete task, (2) number of user errors (tracked on a standardized paper measure by the moderator), and (3) correct entry of data when requested by the app. Improvements across these measurements were hypothesized to occur across attempts (eg, logging a food item attempt 1 versus attempt 2). Satisfaction and general usability were measured via self-reported questionnaires (please see Measures section).

Participants were brought to a private area of the main camp building to complete testing. Testing was conducted by research and graduate assistants with experience in interviewing AYA-SBs. A think-aloud protocol was used [30]; however, the majority of participants did not say what they were thinking during the usability tasks. To avoid distracting the participants, those who did not speak during tasks were given time to share any relevant thoughts after each interaction. Before the testing of My Diet Coach, participants engaged in a card-sorting task to identify barriers to the use of apps for health management. The card sorting results are reported elsewhere [13]. Next, participants were verbally queried about their typical platform for accessing apps (ie, iOS or Android) and then provided a description of My Diet Coach from the Google Play Store (ie, “My Diet Coach helps you find your inner motivation, stay on track, make healthy lifestyle changes, resist food cravings, and avoid exercise laziness and other weight loss difficulties.”). Participants were then provided an Android phone (Moto G5s Plus, 5.5-inch screen). If they were unable to utilize this device because of vision or motor dexterity problems, they were offered the option to complete the testing on an iPad (6th generation), which was encased in a handle-stand cover to improve the participant’s ability to hold the device. The verbal instructions for the first task were as follows:

Now, please imagine that you want to eat healthier. You see this description of My Diet Coach on the Google Play store and decide to download it. Let’s pretend that you just ate one piece of pepperoni pizza for lunch. Please open the app, which is already on the home page, and select “the lightning bolt” to log the pizza you just ate. Feel free to say out loud what you are thinking while you do this. Tell me when you are finished.

This interaction was timed and audiotaped, and any observed user errors or alternative paths to complete the task were noted by the research or graduate assistant. If a participant stopped working on the task, they were prompted with “What’s going through your mind right now?”, followed by “Please do your best to complete the task and let me know when you are finished.” To avoid participant frustration, the task was ended if a user stopped working for 90 consecutive seconds. Once the task was completed, participants were asked to share their thinking with regard to alternative paths taken to complete the task. Participants completed 3 more tasks following this same methodology: (1) exercise (ie, entering a 30-min activity of their choice), (2) second food (ie, entering in eating an apple for a snack), and (3) second exercise (ie, entering a different 15-min activity of their choice). Finally, participants were asked open-ended questions about (1) their impressions of the app (eg, “What are your overall impressions of the logging features of My Diet Coach?” and “Is there anything that you feel is missing?”) and (2) the designs of technology more generally for AYA-SBs (eg, “How could technology work better for you in terms of managing your health?”).

#### Data Collection Approaches

The following traditional data collection methodologies that have been used in the testing of other apps [19,31,32] were selected to evaluate My Diet Coach for AYA-SBs: (1) audio recordings of the testing, (2) standardized interview questions, (3) providing the option for the research/graduate assistants to prompt participants following specific behaviors, (4) validated questionnaires (see Measures section), (5) timing of the tasks with a stopwatch, and (6) research/graduate assistant recording of errors or path deviations (on a standardized paper form).

### Measures

All measures were administered following completion of the interactions with My Diet Coach. Participants were given the opportunity to answer questionnaires via paper and pencil or via an electronic version administered through Opinio [33], licensed and administered by Loyola University Chicago.

#### Demographics

Participants were asked to report the following information: age, sex, race/ethnicity, and SB characteristics, including type, shunt status, and lesion level. Full Scale Intelligence Quotient (FSIQ) was measured and collected for those who also participated in another camp-based study [24-26].

#### System Usability Scale

The System Usability Scale (SUS) is a 10-item self-reported instrument measuring a user’s rating of a product’s usability [34]. Items are rated on a 5-point Likert scale (ie, 1=strongly disagree to 5=strongly agree). Total scores are derived by converting the responses (ie, subtracting 1 from odd-numbered items and 5 from even-numbered items), summing the converted numbers, and multiplying the total by 2 and a half. Although this scoring method yields total scores ranging from 0 to 100, this number is not meant to be interpreted as a percentage. A SUS total score of 68 is considered the cut point for an *average* score or grade of *C*; higher scores are considered above average and lower scores are considered below average [35]. The SUS has been utilized in previous research with youth and adults with SB [36] and had adequate reliability in the current sample (alpha=.86).

#### After-Scenario Questionnaire

The After-Scenario Questionnaire (ASQ) is a 3-item self-reported instrument measuring a user’s satisfaction with a product [37]. Items are rated on a 7-point Likert scale (ie, 1=strongly agree to 7=strongly disagree). Respondent answers to the items are averaged to create a total score, with higher scores indicating higher dissatisfaction following a specific task. To the best of our knowledge, this is the first use of the ASQ with a sample of AYA-SBs. The ASQ demonstrated an adequate Cronbach alpha coefficient (.66) for this sample.

#### Health Questionnaire

The Health Questionnaire is a modified and abbreviated (17 out of 87 original items) version of the 1999 Youth Risk Behavior Survey by the Centers for Disease Control and Prevention (CDC) [38]. Items used for this study address self-reporting of weight, height, desire to change weight, diet, food, and exercise questions appropriate for youth with SB. Data from the Health Questionnaire include categorical (ie, “Which of the following are you trying to do about your weight? Lose weight; Gain weight; Stay the same weight; or I am not trying to do anything about my weight”) and continuous (ie, frequency of behavior) variables. Responses on the Health Questionnaire were used to calculate body mass index (BMI) based on self-reported height and weight, which were calculated using the CDC BMI calculators for children and teens [39] and adults [40]. However, some missing data for BMI were anticipated, as there are established difficulties in obtaining valid measurements of height and weight in people with physical disabilities [41]. The Health Questionnaire was administered to characterize the current health and weight management behaviors of the current sample and has previously been used in studies involving AYA-SBs [42].

### Data Analysis

The demographic and SB characteristics (eg, type of SB), usability testing measurements (eg, time to complete and number of user errors), and questionnaire data were analyzed using descriptive statistics. The *t* test was used to compare differences between the first and second attempts of tasks (eg, time to complete the first food entry compared with time to complete the second food entry). All analyses were run in Statistical Package for the Social Sciences version 24 (IBM Corp), with the 0.05 type I error rate.

## Results

### Participants

A total of 29 participants agreed to participate; however, 1 participant only completed questionnaires as she reported feeling too overwhelmed from the card sorting task [13] to complete usability testing. Participants were primarily young adults (mean 18.11, SD 4.55), female (59%, 17/29), and non-Hispanic white (72%, 21/29), with myelomeningocele (69%, 20/29), with a lumbar (41%, 12/29) or unknown lesion level (41%, 12/29), and with a shunt (79%, 23/29). The BMI of the sample ranged from underweight to obese, with the average BMI falling within the normal range (mean 21.84, SD 4.19). It should be noted that 7 participants (24%) did not have BMI data because of difficulty in reporting current height and/or weight (obtaining accurate height and weight measurements in people with disabilities can pose challenges) [41]. The majority of the sample endorsed wanting to lose (39%, 11/28; one participant did not answer) or maintain (32%, 9/28; one participant did not answer) weight. Table 1 displays the demographic, SB, and health characteristics.

**Table 1:** Demographic, spina bifida, and health characteristics (N=29).

Characteristics		Adolescents and young adults with spina bifida
Age (years), mean (SD); range		18.11 (4.55); 13-30
**Sex,** **n** **(%)**		
	Male	12 (41.4)
	Female	17 (58.6)
**Race/ethnicity,** **n** **(%)**		
	African American	2 (6.9)
	Asian	2 (6.9)
	Caucasian	21 (72.4)
	Hispanic	4 (13.8)
	Other	—^a^
**Spina bifida type,** **n** **(%)**		
	Myelomeningocele	20 (69.0)
	Other	9 (31.0)
	Meningocele	—
	Lipomeningocele	1 (3.4)
	Occulta	—
	Unsure	8 (27.6)
**Lesion level,** **n** **(%)**		
	Thoracic	1 (3.4)
	Lumbar	12 (41.4)
	Sacral	4 (13.8)
	Unsure	12 (41.4)
Shunt present, n (%)		23 (79.3)
Full Scale Intelligence Quotient^b^, mean (SD); range		84.67 (19.51); 55-132
**Personal mobile device,** **n** **(%)**		
	Android	10 (34.5)
	iOS	18 (62.1)
	Did not report	1 (3.4)
Body mass index^c^, mean (SD); range		21.84 (4.19); 14.50-32.00
**Current weight change attempts,** **n** **(%)**		
	Gain	6 (21.4)
	Lose	11 (39.3)
	Maintain	9 (32.1)
	No attempts to change	2 (7.1)

^a^Not applicable.

^b^Data missing for 5 participants because of not participating in the larger camp-related intervention.

^c^Data missing for 7 participants because of not responding about height and/or weight.

#### Health Behaviors

To contextualize the usability outcomes within the sample’s health behaviors, participants reported their current frequency of healthy food consumption, physical activity, screen time, and sleep. Tables 2 to 4 display the health behavior frequencies of the current sample. For dietary behaviors, the CDC recommends a daily minimum intake of (1) 2 fruits/100% juice servings, (2) 2 and a half servings of vegetables, and (3) 3 servings of milk/dairy [43]. Consistent with previous reports of health behaviors in AYA-SBs [42], the majority of the sample reported consuming fruits/100% juice servings (86%, 23/28), vegetables (82%, 22/28), and milk/dairy (89%, 25/28) below the recommended frequencies. For physical activity, teens younger than 18 years are recommended to get at least 60 min of physical activity daily; adults 18 years and older are recommended to get at least 150 min of physical activity weekly (including aerobic, muscle strengthening, and bone strengthening activities) [43]. Given that only (1) 21% (6/28) of the sample endorsed engaging in at least 30 min of strenuous exercise daily, (2) 14% (4/28) endorsed engaging in at least 30 min of nonstrenuous exercise daily, and (3) 4% (1/28) endorsed daily strength exercises, it is likely that the sample is falling short of CDC recommendations for physical activity. However, falling in line with current recommendations of 2 hours or less of screen time per day [44], the majority (69%, 17/26) of the sample reported engaging in 2 hours or less of screen time on weekdays (No time: 11.5%, 1 hour: 11.5%, <1 hour: 11.5%, 2 hours: 34.6%, 3 hours: 15.4%, 4 hours: 3.8%, >5 hours: 11.5%). Finally, and contrary to previous findings of youth and AYA-SBs [42,45],exactly half of the sample endorsed typically sleeping 8 to 9 hours per night [46] and having no or very little difficulty falling and staying asleep.

**Table 2:** Health behavior frequencies.

Frequency of daily healthy food intake and screen time	No times	1-3 times in past 7 days	4-6 times in past 7 days	Once per day	Twice per day	3 times per day	>4 times per day
100% fruit juice^a^ (%)	17.9	35.7	10.7	21.4	7.1	7.1	0
Fruit (%)	17.9	28.6	17.9	17.9	17.9	0	0
Vegetables (%)	14.3	32.1	21.4	10.7	3.6	0	17.9
Milk (%)	25.0	17.9	14.3	17.9	14.3	3.6	7.1

^a^Frequencies reported from adapted version of the Youth Risk Behavior Surveillance System, as reported in the study by Kolbe et al [38].

**Table 3:** Frequency of physical activity.

Physical activity	0 days	1 day	2 days	3 days	4 days	5 days	6 days	7 days
≥30 min of strenuous exercise^a^ (%)	7.1%	10.7	14.3	17.9	10.7	14.3	3.6	21.4
≥30 min of nonstrenuous exercise (%)	21.4	14.3	17.9	10.7	14.3	3.6	3.6	14.3
Strength exercises (%)	53.6	7.1	14.3	3.6	10.7	3.6	3.6	3.6

^a^Frequencies reported from adapted version of the Youth Risk Behavior Surveillance System, as reported in the study by Kolbe et al [38].

**Table 4:** Sleep quality.

Ability to fall asleep	Not at all	Very little	Moderately often	Very often	Almost always
Trouble falling asleep^a^ (%)	26.9	42.3	15.4	7.7	7.7
Trouble staying asleep^a^ (%)	50.0	34.6	11.5	3.8	1.2

^a^Two participants did not report their sleep information.

#### Equipment

The majority of the sample reported using iOS (62%, 18/28) for their personal mobile devices; however, most of them denied using apps to help manage their SB in any way (75%, 21/28). Most participants completed the usability tasks on the Android mobile phone (86%, 24/28); however, 4 participants requested to use the iPad to complete testing because of vision or motor dexterity issues. Given the small sample size and uneven number in the groups, Mann-Whitney U tests were run to compare those who reported owning an iPhone with those who reported owning an Android on the task measurements (ie, completion and error rates and time to complete tasks) to ensure that the device platform did not impact the results. There was no evidence to suggest significant differences between those who owned an iPhone and an Android (*Ps*>.09), with the exception of time to complete the second exercise entry (*P*=.04). For this task, those who owned an iPhone (mean 38, SD 32) were significantly faster than those who owned an Android (mean 58, SD 29). Therefore, the data suggest that it is unlikely that those who owned an iPhone were at a disadvantage if they completed the tasks on an Android device.

### Usability of My Diet Coach for Adolescents and Young Adults With Spina Bifida

#### Completion

About two-thirds of participants (n=20) were able to independently complete a food entry on their first attempt (ie, pizza), with an increase in completion on the second attempt (ie, apple; n=22). For activity entries, about two-thirds of participants (n=20) were able to independently complete an entry on the first attempt, with another increase in completion on the second attempt (n=24). Significance testing was not performed on the completion rates because of lower than expected counts in at least one cell of the contingency table for both activities. The activity entries could be selected by the user, with the most frequently chosen activities being lifting weights, sled hockey, and walking/running. Most participants (86%) were able to come up with their own activities without suggestions from the research or graduate assistant moderating the sessions.

#### Time

Participants significantly decreased their time to complete tasks across the 2 attempts for both food entries (mean time 2:00, SD 1:38 vs mean 1:02, SD :56; *P*=.01) and activity entries (mean time 1:11, SD :50 vs mean :45, SD :31; *P*=.002).

#### Errors and Deviations

The most common user errors involved (1) entering incomplete or inaccurate data (16 out of 112 attempted tasks, 14.3%), (2) being unsure of how to proceed to the next step without being able to recover and complete the task (ie, a *fatal error*; n=13, 11.6%), or (3) believing the task to be complete when it was not (n=8, 7%). The most common deviation was accidentally selecting an option to upgrade the app (n=5, 5%). There was no evidence to suggest a significant difference in the number of errors or deviations across food entry attempts (mean 2.42, SD 2.09 vs mean 1.88, SD 2.14; *P*=.09). However, a decrease in errors or deviations from the expected path across activity entries occurred (mean 1.46, SD 1.96 vs mean 0.58, SD 0.76; *P*=.03).

#### Usability and Satisfaction

Participants completed the SUS and ASQ following their interactions with My Diet Coach to evaluate usability and satisfaction, respectively. SUS ratings for My Diet Coach were highly variable (range 2.50-100). The average SUS rating was 64.17 (SD 29.59; a *below average* SUS score). The average ASQ rating for My Diet Coach was 5.55 (SD 1.36; total scores range from 3 to 21, with higher scores indicating greater dissatisfaction).

#### Sensitivity Analyses by Age

Given the wide range of AYA-SB participants (13-30), sensitivity analyses were conducted to explore any differences that were driven by age. Specifically, exploratory chi-square and *t* tests were conducted to compare demographic and SB-related factors, phone usage, and usability outcomes for those aged 17 years and younger (n=17) with those aged 18 years and older (n=11). There was no evidence to suggest differences in FSIQ, sex, race/ethnicity, type of SB, lesion level, shunt status, type of phone ownership, device used for testing, questionnaire responses, or any usability testing outcomes (*Ps*≥.1), with the exception of adolescents being more likely to have successfully completed the first exercise entry (*P*=.03).

#### Qualitative Feedback

Although the usability tasks were audiotaped, the majority of participants did not engage in the suggested *think-aloud* method of completing tasks. Therefore, qualitative feedback occurred through responses to structured questions that were administered to all participants after completing the usability testing (please see Procedures section).

Despite the variable usability ratings, the majority of participants stated that My Diet Coach would be useful for them (64%, 18/28). When queried as to why this app might be useful, the most common response was related to the app reminding and encouraging them to change their eating and activity choices. For example:

I could see how many calories I'm burning and consuming, and balance those. I definitely see me using in the future.

17-year-old female

[This app] would help me make healthy choices.

17-year-old female

It would help me realize I need more fruits and vegetables.

21-year-old male

Although the current usability testing evaluated initial learnability, qualitative feedback also suggested that learnability might improve with long-term use (eg, “It was a little tricky at first. As I used it more, it became easier to use.” [13-year-old male]; “It seems pretty easy to use once you get used to it.” [18-year-old female]). Suggested improvements specific to AYA-SBs included (1) tutorials (eg, “Make it more self-explanatory. Have practice stuff, give step-by-step directions to enter stuff.” [14-year-old female] and “There’s a lot of information in it with no instructions.” [20-year-old female]), (2) fewer logging options and/or simplifying the design throughout (eg, “It’s too confusing with too many steps” [17-year-old female] and “Make it easier by not making it so heavy in content and choices.” [19-year-old female]), (3) more activity options for those who ambulate by wheelchair (eg, “It needs to add exercise for people in wheelchairs.” [23-year-old male]), and (4) notifications to prompt use (eg, “I need reminders [to do this] on my phone…Remind me to do my exercises and eat healthy.” [20-year-old female]).

## Discussion

### Principal Findings

The purpose of this study was to evaluate the usability attribute of learnability for My Diet Coach, a publicly available, high-quality app [18] designed for the general public, for AYA-SBs. The majority of the sample endorsed wanting to maintain or lose weight and reported dietary and exercise behaviors that fell short of the CDC recommendations [43]. These characteristics, combined with the increased risk for obesity in people with SB [3], make this app a potentially appropriate tool for aiding in weight management for this sample. The majority of the sample was able to complete the tasks of entering foods and exercises into the app, with improved performance on the second attempts for both tasks. Consistent with the hypotheses, AYA-SBs were able to complete initial tasks with the app in under 5 min [19,20] and demonstrated decreased task time on the second attempts. Despite this success, but also consistent with our hypotheses, high variability of usability was observed, suggesting that added tutorial features for users with special needs might be beneficial. To the best of our knowledge, there is no established metric for the ideal number of user errors on initial interactions with an app [47,48]; however, the error rates (1) appeared high for a limited number of required task actions and (2) raise questions about the initial learnability of this app for subsets of AYA-SBs. Finally, responses to validated usability questionnaires and open-ended interview questions suggest variable usability for this group and also emphasize the importance of querying AYA-SBs in a mixed-methods fashion.

A user needs model for AYA-SBs was recently created for BITs aiming to improve self-management [16]. This user needs model was framed within the BIT model, which includes conceptual (*why* the BIT is needed and *how* it may achieve such aims) and technical (*what* is delivered to BIT users and *how* and *when* the delivery may occur) aspects for designing and deploying BITs [49]. In utilizing a high-quality app already publicly available (ie, My Diet Coach), this study evaluated the usability of existing BIT elements (ie, *what* is delivered) and characteristics (ie, *how* the delivery may occur) for AYA-SBs. The findings suggest that in applying the user needs model for AYA-SBs to the design of BITs specific to this population, design teams do not need to *reinvent the wheel* and create entirely new BITs for AYA-SBs (a cost-heavy venture from a financial and time perspective). Indeed, it appears that the majority of AYA-SBs in the current sample can independently learn to complete specific data entry tasks within a reasonable time frame using an existing BIT targeting weight management [19,20].

Although the current findings suggest that My Diet Coach is learnable for AYA-SBs, it also implicates personalizing existing elements and characteristics for AYA-SBs and related users (eg, youth with physical and/or motor disabilities) [16]. The evaluated elements of My Diet Coach involved active, text-based data entry. Consistent with the literature on AYAs with similar symptom profiles [16], the AYA-SB participants reported that these elements could be improved for their use by having tutorials, being simpler, and having less logging options. Possibly because of the lack of such features and/or the executive functioning problems associated with SB [23], participants also displayed several errors for a relatively simple data entry path. In addition, the entry options were not optimized for variability in accessibility (eg, for those who ambulate with leg braces or by wheelchair). Therefore, although AYA-SBs demonstrated that they are capable of using elements featuring text-based data entry, they may be unlikely to persist in using such elements in real-world conditions. Moreover, the data collected might not be as accurate as compared with the use of other means of collection (eg, voice-to-text entry or passive data collection via an accelerometer). These findings suggest that a multisensory and multimethod approach to BIT elements is likely warranted for AYA-SBs [16] but that text-based data entry is a learnable element for AYA-SBs.

Given the variable levels of impairment in motor dexterity, coordination, hearing, vision, and visuospatial processing in people with SB [23], BIT characteristics likely also need to demonstrate flexibility. My Diet Coach demonstrated multiple platform capabilities in testing, which was important and necessary for AYA-SBs (ie, participants accessed the app via an Android phone or an iPad). The text-based data entry tasks involved elements of the app that had limited graphics, which falls in line with user needs for simple and/or limited graphics [16] because of posterior attention difficulties (which impact the ability to focus and shift attention) [23]. My Diet Coach is also designed to include customized reminders around user goals (ie, “Drink water” and “Always be prepared with healthy snacks”). This characteristic was reported as desired following testing and also falls in line with the user needs model [16]. However, it was not evaluated in this study. Therefore, the design characteristics of My Diet Coach appeared to align well with the needs of AYA-SBs, but future research will need to evaluate how usable such characteristics are when users are confronted with other common tasks (eg, interacting with notification reminders).

The majority of participants were able to complete the tasks and stated that the app would be personally useful. However, questionnaire responses were not indicative of high usability and satisfaction, and the majority of the sample reported that they do not use apps to manage their SB (75%). These discrepancies may have multiple explanations. First, young people with SB have variable cognitive profiles, with many falling within the category of having a mild intellectual disability [2]. The differences between qualitative and quantitative feedback may reflect the importance of feedback methods when assessing AYA-SBs. It also highlights the need for continued validation of usability measures for individuals with disabilities and/or special needs. Second, AYA-SBs may use technology less frequently and/or in selective ways compared with the general population [50]. It is likely that BITs may sound appealing in theory but are anticipated to have multiple barriers (being unintuitive, not specialized for the needs of people with SB, etc) or are in conflict with time already allotted to TV viewing or social networking [3,13]. Finally, it is possible that participants believed that the app had been developed by those conducting the usability testing. Therefore, qualitative feedback may have been driven by a desire to please the evaluators.

### Limitations

This study builds upon previous work establishing the importance and feasibility of conducting usability testing with AYA-SBs [51,52]. However, the findings should be considered in light of specific limitations. First, the sample was recruited from the YMCA-sponsored Camp Independence [24-26]. The sample consisted of a wide age range of AYA-SBs who have the support and ability to attend a sleep-away summer camp session and who were also primarily non-Hispanic whites. More usability testing of apps for self-management is required, targeting AYA-SBs with greater diversity and within more *real-world* conditions. Second, testing was focused solely upon data entry of monitoring behaviors that are common in weight management strategies (ie, food intake and physical activity monitoring). Furthermore, the usability tasks were designed to be brief (ie, 4 entry tasks that took an average of about 2 min per task), so as to not keep participants from the activities of the camp for a significant amount of time. It is unclear how the current findings extend to (1) the entry and monitoring of other behavioral change strategies, (2) long-term use, and (3) other types of app elements and other apps, more generally. Third, the majority of participants reported using iOS for their personal devices, yet the majority opted to complete usability testing on an Android device. Comparisons of usability outcomes across these 2 groups did not suggest a disadvantage for iPhone users. However, to avoid this confound in future research, facilitating acclimation to the operating system before usability testing would be ideal. Finally, it is unclear if the presence of the research/graduate assistants facilitating the testing sessions had any impact on performance.

### Conclusions

For clinicians and engineers designing BITs for pediatric and AYA populations, the findings of this study suggest that it may be possible to iterate from existing technologies to personalize apps for specific populations. Doing so may decrease both the financial and time burden associated with designing and building a new technology. However, the use of appropriate user-centered design principles and the use of formative usability testing is still critical [49,53]. Indeed, AYA-SBs were able to learn how to complete specific tasks independently on a weight management app, but design changes consistent with previously proposed user needs are still recommended [16].

## Abbreviations

ASQ: After-Scenario Questionnaire
AYA: adolescents and young adults
AYA-SB: adolescents and young adults with spina bifida
BIT: behavioral intervention technology
BMI: body mass index
CDC: Centers for Disease Control and Prevention
FSIQ: Full Scale Intelligence Quotient
SB: spina bifida
SUS: System Usability Scale
YMCA: Young Men’s Christian Association
